# Antimicrobial Susceptibility Profiles of Extended‐Spectrum *β*‐Lactamase‐Producing *Escherichia coli* Isolated From Intensively Reared and Free‐Range Chickens in Selected Districts of Zambia

**DOI:** 10.1002/vms3.71090

**Published:** 2026-07-16

**Authors:** Kenneth Chawinga, Bernard M. Hang'ombe, Geoffrey Mainda, Francis Mbaimbai, Liywalii Mataa, Emmanuel Kabwali, Fusya Y. Goma, Geoffrey Muuka, Musso Munyeme, Rajhab Sawasawa Mkakosya, Isaac Thom Shawa

**Affiliations:** ^1^ Kamuzu University of Health Sciences Blantyre Malawi; ^2^ School of Veterinary Medicine University of Zambia Lusaka Zambia; ^3^ Department of Veterinary Services Ministry of Fisheries and Livestock Government of the Republic of Zambia Lusaka Zambia; ^4^ Food and Agriculture Organization of the United Nations Lusaka Zambia; ^5^ School of Natural Resources Copperbelt University Kitwe Zambia; ^6^ Biomedical and Forensic Sciences Department University of Derby Derby UK

**Keywords:** antimicrobial resistance, bla*_CTX‐M_*, ESBL‐producing *E. coli*, free‐range chickens, intensively reared chickens, multidrug resistance, multiplex PCR, One Health, Zambia

## Abstract

**Background:**

Extended‐spectrum *β*‐lactamase (ESBL)‐producing *Escherichia coli* in poultry is an emerging One Health concern, yet comparative data on intensive versus free‐range systems in sub‐Saharan Africa remain sparse.

**Objectives:**

To compare the prevalence, susceptibility profiles and *β*‐lactamase gene content of ESBL‐producing *E. coli* from intensively reared and free‐range chickens in Central Province, Zambia.

**Methods:**

In a cross‐sectional study (March–April 2021), 112 pooled faecal samples (56 per production system; 10 birds per sampling point) were collected across seven veterinary camps in Kabwe and Kapiri Mposhi districts and processed within 24 h. Presumptive ESBL *E. coli* was isolated on MacConkey agar with 2 mg/L cefotaxime and identified biochemically. Susceptibility to 11 antimicrobials was tested by Kirby–Bauer disc diffusion per CLSI M100 (2020), and ESBL production was confirmed by combination‐disc synergy. *bla_CTX‐M_*, *bla_TEM_* and *bla_SHV_* were detected by multiplex PCR. Proportions were compared using Fisher's exact test with 95% Wilson confidence intervals (CIs).

**Results:**

ESBL‐producing *E. coli* was recovered from 13/112 pooled samples (11.6%, 95% CI: 6.9%–18.9%), with no difference between intensively reared (7/56, 12.5%) and free‐range (6/56, 10.7%) chickens (*p* = 1.00). All 13 isolates were multidrug‐resistant, with the highest resistance to tetracycline and cefotaxime (both 100%), streptomycin (84.6%) and sulfamethoxazole–trimethoprim (69.2%). *bla_CTX‐M_* was present in all 13 isolates, *bla_TEM_* in 4 (30.8%) and *bla_SHV_* in 1 (7.7%), with co‐carriage in 5 isolates.

**Conclusions:**

Multidrug‐resistant, *bla_CTX‐M_*‐driven ESBL *E. coli* circulates at comparable levels in both production systems, consistent with environmental dissemination. Integrated One Health surveillance and antimicrobial stewardship are warranted.

## Graphical abstract

Multidrug‐resistant, *bla_CTX‐M_*‐driven ESBL *E. coli* circulates at comparable levels in intensively‐reared and free‐range chicken production systems, consistent with environmental dissemination. One Health surveillance and antimicrobial stewardship are warranted.

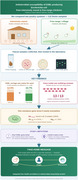

## 1 Introduction

Antimicrobial resistance (AMR) is a globally recognised public‐health threat, yet comprehensive data on antimicrobial usage, the prevalence of resistance and its downstream impact on human health remain limited in many low‐ and middle‐income countries (Jenkins 2015; Ahmed et al. 2024). Poultry production is increasingly recognised as an important node for the selection, amplification and dissemination of antimicrobial‐resistant *Escherichia coli* in both community and environmental compartments (van den Bogaard and Stobberingh 2001; Dierikx et al. 2013).

Poultry is a central pillar of Zambia's livestock industry. The most recent national livestock census estimated the poultry population at approximately 94 million broilers, 5.8 million layers and 15 million village (free‐range) chickens (Ministry of Fisheries and Livestock 2023). The sector faces substantial infectious‐disease burdens, with *Salmonella* spp., *Campylobacter* spp. and *E. coli* being among the most frequently recovered bacterial pathogens, alongside rising concerns about resistance to first‐line antimicrobials (Ziba et al. 2020; Phiri et al. 2020; Rouger et al. 2017).

As in many African settings, poultry meat and eggs are an affordable and accessible source of animal protein for Zambian households (Musaba and Mseteka 2014), and safeguarding their microbiological quality is therefore a public‐health priority. The dual pressures of disease burden and market demand incentivise the prophylactic and growth‐promoting use of antimicrobials, which is a well‐documented driver for the emergence of resistance (Hedman et al. 2020; Agyare et al. 2019). Inadequate observance of withdrawal periods further compromises food safety through antimicrobial residues in meat and eggs (Gonzalez Ronquillo and Angeles Hernandez 2017).

Extended‐spectrum *β*‐lactamases (ESBLs), and in particular CTX‐M‐type enzymes, have become the dominant mechanism of cephalosporin resistance in *Enterobacterales*, causing serious extra‐intestinal infections in humans and complicating treatment because of their frequent association with resistance to other antimicrobial classes (Bevan et al. 2017; Puspandari et al. 2021). Poultry and poultry environments are increasingly implicated as reservoirs for these enzymes (Chileshe et al. 2024).

In response to the global AMR agenda, Zambia established the Antimicrobial Resistance Coordinating Committee (AMRCC) in 2015 and adopted a National Action Plan on AMR in 2017 (World Health Organization 2017). Nevertheless, the carriage of ESBL‐producing *E. coli* in Zambian poultry, and in particular the comparative carriage in intensively reared versus free‐range systems, has received little empirical attention. This represents a critical evidence gap, because free‐range systems are often assumed to be at lower risk owing to low antimicrobial use, yet environmental reservoirs may sustain resistance irrespective of on‐farm practices.

This study aimed to compare the antimicrobial susceptibility profiles of ESBL‐producing *E. coli* isolated from intensively reared and free‐range chickens in selected districts of Zambia. Specifically, we (i) determined the prevalence of ESBL‐producing *E. coli* carriage in each production system; (ii) characterised isolates phenotypically by disc diffusion against a panel of 11 antimicrobials and (iii) genotyped phenotypically confirmed ESBL isolates for *bla_CTX‐M_*, *bla_TEM_* and *bla_SHV_* by multiplex PCR.

## 2 Materials and Methods

### 2.1 Study Design, Setting and Sampling Frame

**FIGURE 1:**
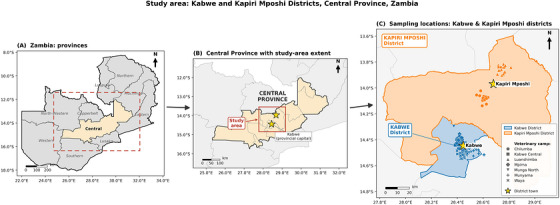
Study area showing Kabwe and Kapiri Mposhi Districts in Zambia's Central Province. (A) Map of Zambia indicating the Central Province (highlighted) and the study area (red box). (B) Detailed map of Kabwe and Kapiri Mposhi Districts showing sampling locations within veterinary camps. Administrative boundaries are based on data from the Zambia Central Statistical Office/OCHA ROSEA (2020). Coordinate reference system: WGS84 (EPSG:4326).

A cross‐sectional, stratified study was conducted in the Kabwe and Kapiri Mposhi districts of Central Province, Zambia (Figure 1), between March and April 2021. The sampling frame was defined by veterinary camp administrative boundaries and was stratified by two criteria: (i) District and (ii) production system (intensively reared vs. free‐range). Sample size was informed by established methods for comparing proportions between two groups (Persoons et al. 2011) and was operationalised by recruiting 56 households/farms per production system (112 in total). At each sampling point, 10 individual freshly voided faecal droppings were pooled to form one composite sample, providing a flock‐level representation (Nene et al. 2024).

Seven veterinary camps participated: Five in Kabwe District (Munga North, Waya, Mpima, Kabwe Central and Munyama) and two in Kapiri Mposhi District (Chilumba and Luanshimba). The distribution of samples across districts, veterinary camps and production systems is summarised in Table 1. Kabwe District contributed 77 samples (68.75%, 95% CI: 59.8%–76.3%) and Kapiri Mposhi contributed 35 samples (31.25%, 95% CI: 23.7%–40.2%).

**TABLE 1:** Distribution of 112 pooled faecal samples by district, veterinary camp and poultry production system.

District	Veterinary camp	Samples (*n*)	Proportion of total, % (95% CI)	Intensive ESBL+/*n*	Free‐range ESBL+/*n*	Total ESBL + *n* (%)
Kabwe	Munga North	1	0.9 (0.2–4.9)	0	0	0
Kabwe	Waya	17	15.2 (9.7–23.0)	1	0	1
Kabwe	Mpima	12	10.7 (6.2–17.9)	2	1	3
Kabwe	Kabwe Central	32	28.6 (21.0–37.5)	2	0	2
Kabwe	Munyama	15	13.4 (8.3–20.9)	0	2	2
Kapiri Mposhi	Chilumba	13	11.6 (6.9–18.9)	1	1	2
Kapiri Mposhi	Luanshimba	22	19.6 (13.3–28.0)	1	2	3
Kabwe	Subtotal (5 camps)	77	68.8 (59.8–76.3)	5	3	8
Kapiri Mposhi	Subtotal (2 camps)	35	31.3 (23.7–40.2)	2	3	5
Total	7 veterinary camps	112	100	7	6	13 (11.6)

*Note*: Each pooled sample comprised 10 individual faecal droppings. Proportions of total are reported with 95% Wilson score confidence intervals. For each veterinary camp the number of ESBL‐positive pooled samples from intensively reared and free‐range flocks is tabulated separately.

Abbreviations: CI, confidence interval; ESBL, extended‐spectrum *β*‐lactamase.

At each veterinary camp, we attempted to collect eight composite samples (four from free‐range and four from intensive systems) to ensure balanced representation. Where an insufficient number of intensive or free‐range farms were available in a given camp, additional sampling was performed in a neighbouring camp within the same district to achieve the target of 56 samples per production system.

### 2.2 Sample Collection and Transport

Freshly voided droppings were aseptically collected with sterile gloves from the dorsal surface to avoid contact with the ground (De Carli et al. 2015). Ten droppings per sampling point were combined into a single sterile sample bag, labelled and placed in a cool‐box on ice packs. Samples were transported to a microbiology laboratory and processed within 24 h of collection; samples were not frozen prior to enrichment.

### 2.3 Isolation and Identification of Presumptive ESBL‐Producing *E. coli*

A 1.0 g portion of each composite sample was added to 9 mL of Luria–Bertani (LB) broth for non‐selective enrichment and incubated at 37°C for 18–24 h. A loopful (∼100 µL) of each enrichment was then streaked onto MacConkey agar supplemented with 2 mg/L cefotaxime (E&O Laboratories Ltd., UK) for selective recovery of presumptive ESBL‐producing Gram‐negative bacteria, and plates were incubated at 37°C for 18–24 h. A single well‐isolated, lactose‐fermenting, pink colony per plate was subcultured for purity and identified biochemically by colony morphology, Gram staining, indole, methyl red, Voges–Proskauer, citrate, triple‐sugar‐iron and urease reactions, according to standard methods (Erkmen 2021). *E. coli* ATCC 25922 was used as a quality‐control strain throughout.

### 2.4 Antimicrobial Susceptibility Testing

Antimicrobial susceptibility testing was performed by the Kirby–Bauer disc‐diffusion method on Mueller–Hinton agar (Becton, Dickinson and Company, Franklin Lakes, NJ, USA). The panel comprised 11 agents from 8 classes: sulfamethoxazole–trimethoprim (co‐trimoxazole; 25 µg), chloramphenicol (30 µg), gentamicin (10 µg), tetracycline (30 µg), ciprofloxacin (5 µg), streptomycin (10 µg), nalidixic acid (30 µg), piperacillin–tazobactam (110 µg), norfloxacin (10 µg), cefotaxime (30 µg) and ceftriaxone (30 µg). Inhibition zones were measured after 16–18 h of incubation at 35 ± 2°C and interpreted using *Clinical and Laboratory Standards Institute (CLSI) M100 Breakpoints* (30th edition, 2020) (Clinical and Laboratory Standards Institute 2020). Multidrug resistance (MDR) was defined as non‐susceptibility to at least one agent in more than or equal to three antimicrobial classes, in line with Magiorakos et al. (2012).

### 2.5 Phenotypic Confirmation of ESBL Production

Phenotypic ESBL production was confirmed using the combination‐disc (double‐disc synergy) test with cefotaxime (30 µg) and ceftazidime (30 µg) discs tested alone and in combination with clavulanic acid (10 µg). A ≥ 5‐mm increase in the zone diameter of either cephalosporin in the presence of clavulanic acid (compared with the cephalosporin alone) was considered confirmatory of ESBL production, in accordance with CLSI M100 guidance (Clinical and Laboratory Standards Institute 2020; Kazemian et al. 2019).

### 2.6 DNA Extraction and Multiplex PCR Detection of ESBL Genes

Genomic DNA was extracted from fresh, overnight colonies by the boiling‐lysis method: one to two colonies were suspended in 500 µL of nuclease‐free water, heated at 100°C for 10 min, centrifuged at 13,000 × *g* for 2 min, and the supernatant (containing template DNA) was used directly in PCR (Dashti et al. 2009).

A multiplex PCR targeting *bla_CTX‐M_*, *bla_TEM_* and *bla_SHV_* was performed using primers listed in Table 2, as previously described (Ranjan et al. 2015; Mahmud et al. 2020). Each 20 µL reaction contained 10 µL of 2 × Phusion Flash High‐Fidelity Master Mix (Finnzymes Oy, Espoo, Finland), 0.5 µL of each forward and reverse primer (10 µM final concentration per primer pair), 1.0 µL of DNA template and nuclease‐free water to 20 µL. Thermal cycling was performed on a conventional block cycler as follows: Initial denaturation at 95°C for 5 min; 30 cycles of denaturation at 95°C for 30 s, annealing at 55°C for 30 s and extension at 72°C for 60 s; and a final extension at 72°C for 10 min. A previously characterised ESBL‐positive *E. coli* strain (provided by an independent veterinary research institution) served as a positive control, and nuclease‐free water was used as a no‐template negative control in every run.

**TABLE 2:** Primers used in the multiplex PCR for detection of blaCTX‐M, blaTEM and blaSHV.

Target gene	Sequence (5′ → 3′)	*T*_m_ (°C)	Amplicon (bp)	Reference
*bla_CTX‐M_*	Fw: CGATGTGCAGTACCAGTAA	55	585	Ranjan et al. (2015)
	Rv: TTAGTGACCAGAATCAGCGG			
*bla_TEM_*	Fw: ATAAAATTCTTGAAGACGAAA	55	1080	Mahmud et al. (2020)
	Rv: GACAGTTACCAATGCTTAATC			
*bla_SHV_*	Fw: GGGTTATTCTTATTTGTCGC	55	930	Mahmud et al. (2020)
	Rv: TTAGCGTTGCCAGTGCTC			

*Note*: Primer sequences were adopted from the indicated references. Multiplex PCR was performed with a common annealing temperature of 55°C.

Abbreviations: bp, base pairs; Fw, forward primer; Rv, reverse primer; Tm, annealing temperature.

PCR products were resolved by electrophoresis on 1% (w/v) agarose gels in 1 × Tris‐acetate‐EDTA buffer containing 0.5 µg/mL ethidium bromide at 90 V for 45 min. A 100 bp DNA ladder (GeneRuler, Thermo Fisher Scientific, Waltham, MA, USA) was included in each run as a molecular‐size marker. Gels were visualised and photographed under ultraviolet light using a Gel Doc XR+ gel‐documentation system (Bio‐Rad Laboratories, Hercules, CA, USA). Expected amplicon sizes were 585 bp (*bla_CTX‐M_*), 1080 bp (*bla_TEM_*) and 930 bp (*bla_SHV_*).

### 2.7 Statistical Analysis

Data were entered in Microsoft Excel (Microsoft Corp., Redmond, WA, USA) and analysed in R v4.3.1 (R Foundation for Statistical Computing, Vienna, Austria). Proportions are reported as percentages with 95% Wilson score confidence intervals (CIs). Comparisons of proportions between intensively reared and free‐range chickens, and between districts, were performed using Fisher's exact test (two‐sided). The threshold for statistical significance was set at *p* < 0.05. All tests were two‐tailed.

## 3 Results

### 3.1 Sampling Distribution

A total of 112 pooled faecal samples were collected from seven veterinary camps in the Kabwe (*n* = 77; 68.75%, 95% CI: 59.8%–76.3%) and Kapiri Mposhi (*n* = 35; 31.25% 95% CI: 23.7%–40.2%) districts. An equal number of samples (56) was obtained from each of the two production systems (intensively reared and free‐range). The distribution across veterinary camps and production systems is presented in Table 1.

### 3.2 Prevalence of ESBL‐Producing *E. coli* and Comparison Between Production Systems

Phenotypically confirmed ESBL‐producing *E. coli* was recovered from 13 of the 112 pooled samples (overall prevalence 11.6%, 95% Wilson CI: 6.9%–18.9%). Stratifying by production system, ESBL *E. coli* was detected in 7 of 56 intensively reared flocks (12.5%, 95% CI: 6.2%–23.6%) and in 7 of 56 free‐range flocks (12.5%, 95% CI: 6.2%–23.6%). The difference between the two systems was not statistically significant (Fisher's exact test: OR 1.19, 95% CI approximated by exact method; *p* = 1.00; Table 3). At the veterinary‐camp level, ESBL‐positive samples were identified in all camps except Munga North (Table 1).

**TABLE 3:** Prevalence of ESBL‐producing *Escherichia coli* by poultry production system and Fisher's exact test for comparison.

Production system	ESBL+	Total (*n*)	Prevalence, % (95% Wilson CI)	Odds ratio	Fisher *p*
Intensively reared	7	56	12.5 (6.2–23.6)	1.19 (reference)	—
Free‐range	6	56	10.7 (5.0–21.5)	0.84	1.00
Overall	13	112	11.6 (6.9–18.9)	—	—

*Note*: ESBL phenotype was confirmed by the combination‐disc (double‐disc synergy) test per CLSI M100 (2020). Proportions are reported with 95% Wilson score confidence intervals. The Fisher's exact test was two‐sided; the odds ratio compares free‐range to intensively reared (reference).

Abbreviation: CI, confidence interval.

### 3.3 Antimicrobial Susceptibility Profile of ESBL Isolates

The 13 phenotypically confirmed ESBL‐producing *E. coli* isolates showed extensive and non‐susceptibility to multiple antimicrobial classes (Table 4). Resistance was universal (100%) to tetracycline and cefotaxime, reflecting both the selective pressure of the isolation medium and the strong association between ESBL genes and tetracycline resistance reported elsewhere (Carattoli 2013). Resistance was also high to streptomycin (84.6%, 95% CI: 57.8%–95.7%), sulfamethoxazole–trimethoprim (69.2%, 95% CI: 42.4%–87.3%), chloramphenicol (61.5%, 95% CI: 35.5%–82.3%) and piperacillin–tazobactam (53.8%, 95% CI: 29.1%–76.8%). Moderate resistance was recorded for gentamicin and nalidixic acid (each 30.8%, 95% CI: 12.7%–57.6%) and ciprofloxacin (23.1%, 95% CI: 8.2%–50.3%). Only 1 of 13 (7.7%, 95% CI: 1.4%–33.3%) and 0 of 13 (0%, 95% CI: 0.0%–22.8%) isolates were resistant to norfloxacin and ceftriaxone, respectively.

**TABLE 4:** Antimicrobial susceptibility profile of the 13 phenotypically confirmed ESBL‐producing *Escherichia coli* isolates, stratified by production system.

Class	Antimicrobial (disc content)	Intensive *n*/7 (%)	Free‐range *n*/6 (%)	All ESBL *n*/13 (%, 95% CI)
Tetracyclines	Tetracycline (30 µg)	7 (100.0)	6 (100.0)	13 (100.0; 77.2–100.0)
Cephalosporins, 3rd gen.	Cefotaxime (30 µg)	7 (100.0)	6 (100.0)	13 (100.0; 77.2–100.0)
Cephalosporins, 3rd gen.	Ceftriaxone (30 µg)	0 (0.0)	0 (0.0)	0 (0.0; 0.0–22.8)
Aminoglycosides	Streptomycin (10 µg)	6 (85.7)	5 (83.3)	11 (84.6; 57.8–95.7)
Aminoglycosides	Gentamicin (10 µg)	2 (28.6)	2 (33.3)	4 (30.8; 12.7–57.6)
Sulfonamide/DHFR‐inhibitor	Sulfamethoxazole–trimethoprim (25 µg)	5 (71.4)	4 (66.7)	9 (69.2; 42.4–87.3)
Phenicols	Chloramphenicol (30 µg)	4 (57.1)	4 (66.7)	8 (61.5; 35.5–82.3)
Penicillin + β‐lactamase inhibitor	Piperacillin–tazobactam (110 µg)	4 (57.1)	3 (50.0)	7 (53.8; 29.1–76.8)
Quinolones	Nalidixic acid (30 µg)	2 (28.6)	2 (33.3)	4 (30.8; 12.7–57.6)
Fluoroquinolones	Ciprofloxacin (5 µg)	2 (28.6)	1 (16.7)	3 (23.1; 8.2–50.3)
Fluoroquinolones	Norfloxacin (10 µg)	1 (14.3)	0 (0.0)	1 (7.7; 1.4–33.3)

*Note*: Percentages are the proportion of resistant isolates within each subgroup. Due to the small size of each subgroup (*n* = 7 intensive; *n* = 6 free‐range), subgroup comparisons are under‐powered and no formal statistical tests are reported; overall prevalences with 95% Wilson CIs are provided in the final column. All isolates (13/13, 100%; 95% CI: 77.2%–100.0%) met the Magiorakos et al. (2012) definition of multidrug resistance (non‐susceptibility to ≥ 1 agent in ≥ 3 classes).

Abbreviations: CI, confidence interval; DHFR, dihydrofolate reductase.

All 13 (100%) ESBL‐producing *E. coli* isolates satisfied the MDR criterion of Magiorakos et al. (2012) (non‐susceptibility to ≥ 1 agent in ≥ 3 antimicrobial classes), with individual isolates expressing resistance to between four and six of the eight classes tested. Given the overall small number of ESBL isolates (*n* = 13) and the pooled‐sampling design, formal per‐antibiotic comparisons between the intensive and free‐range subgroups were underpowered and are therefore reported descriptively in Table 4. Intensively reared flocks yielded 7 of the 13 ESBL isolates (53.8%) and free‐range flocks yielded the remaining 6 (46.2%); the resistance profiles seen in both subsets were qualitatively similar, with tetracycline, cefotaxime, streptomycin and sulfamethoxazole–trimethoprim dominating in each.

### 3.4 Distribution of ESBL Genes

**FIGURE 2:**
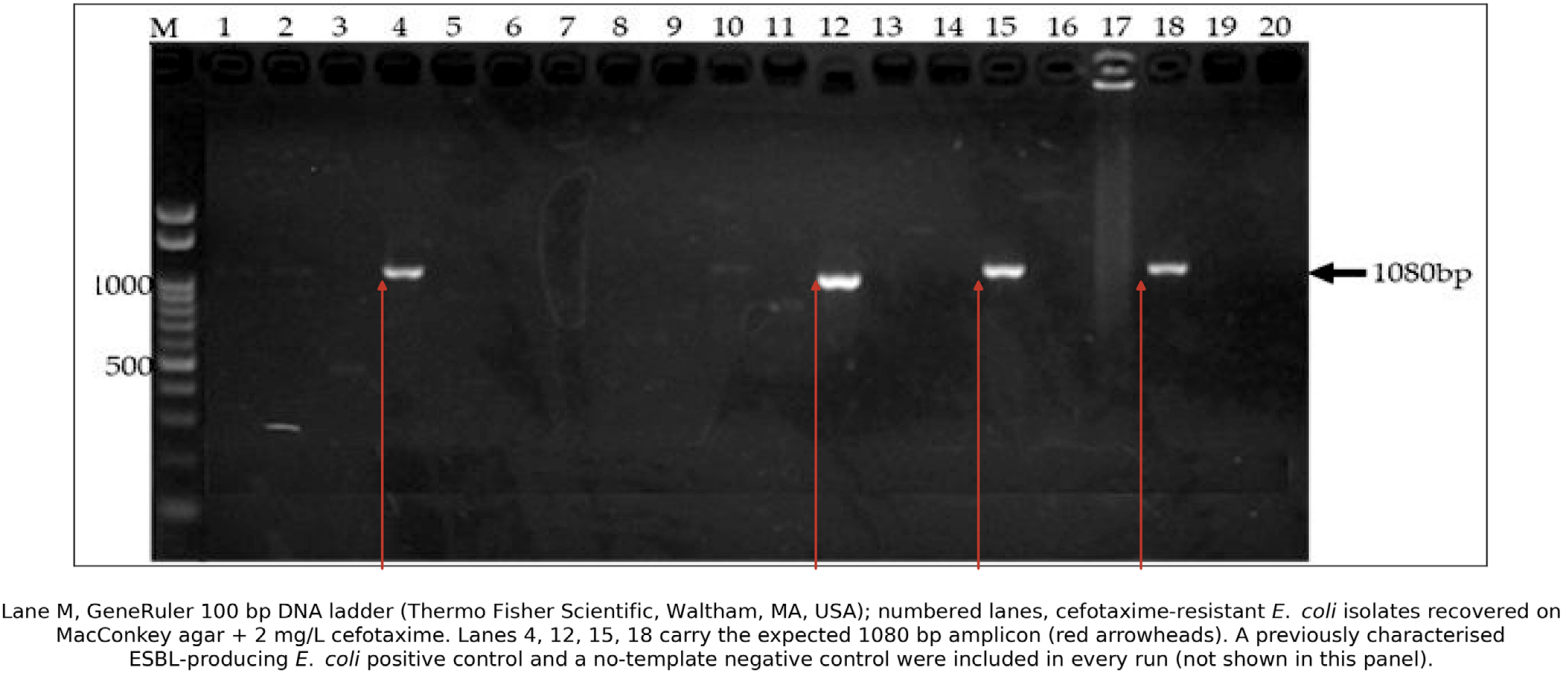
Representative agarose gel electrophoresis (1%, w/v) of the *bla_TEM_* multiplex PCR product (expected amplicon size 1080 bp). Lane M: 100 bp DNA ladder (GeneRuler, Thermo Fisher Scientific); Lane 1: positive control; Lane 2: no‐template (negative) control; Lanes 4, 12, 15 and 18: *bla_TEM_*‐positive isolates (nos. 10, 16, 20 and 21). A very faint non‐specific band may be visible in some lanes and should not be interpreted as a positive amplification at the target size.

**FIGURE 3:**
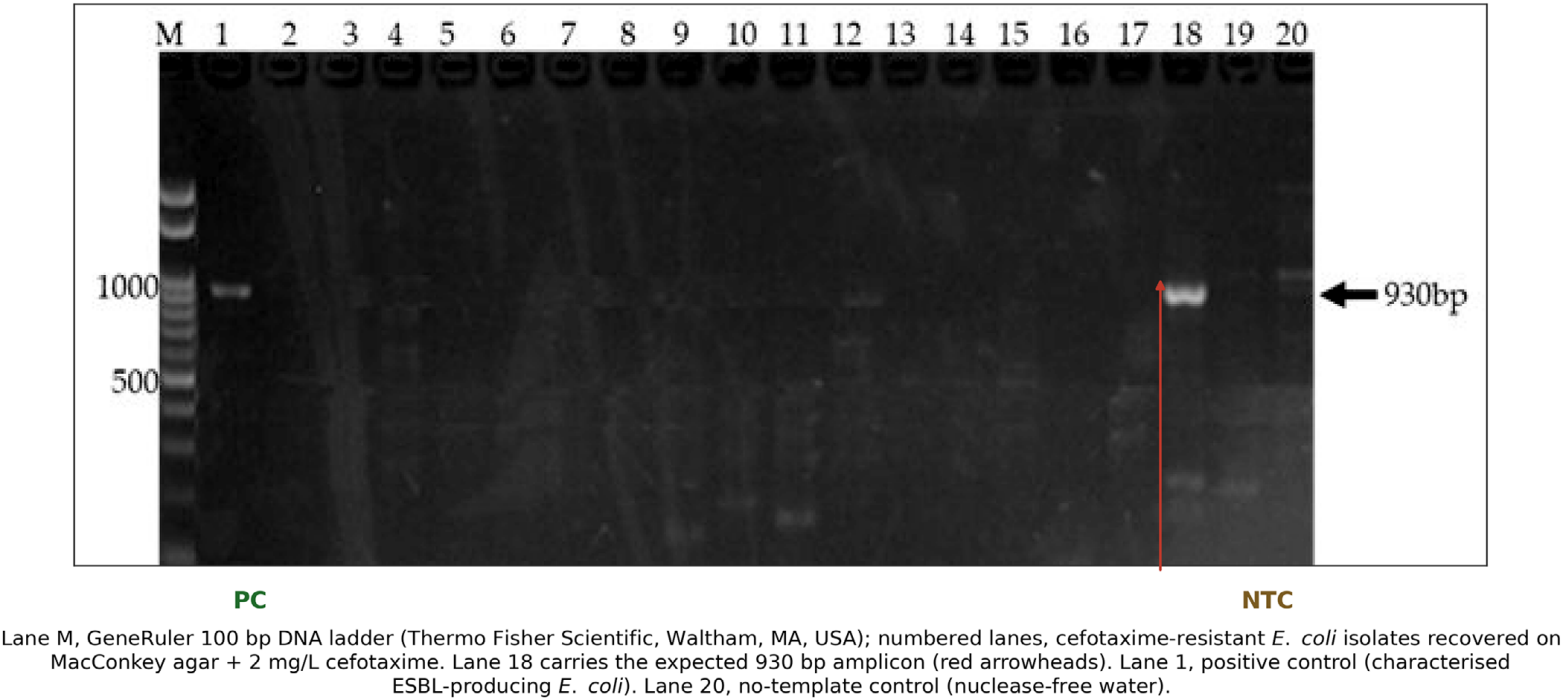
Representative agarose gel electrophoresis (1%, w/v) of the *bla_SHV_* multiplex PCR product (expected amplicon size 930 bp). Lane M: 100 bp DNA ladder; Lane 1: positive control; Lane 2: no‐template (negative) control; Lane 18: *bla_SHV_*‐positive isolate (no. 20).

**FIGURE 4:**
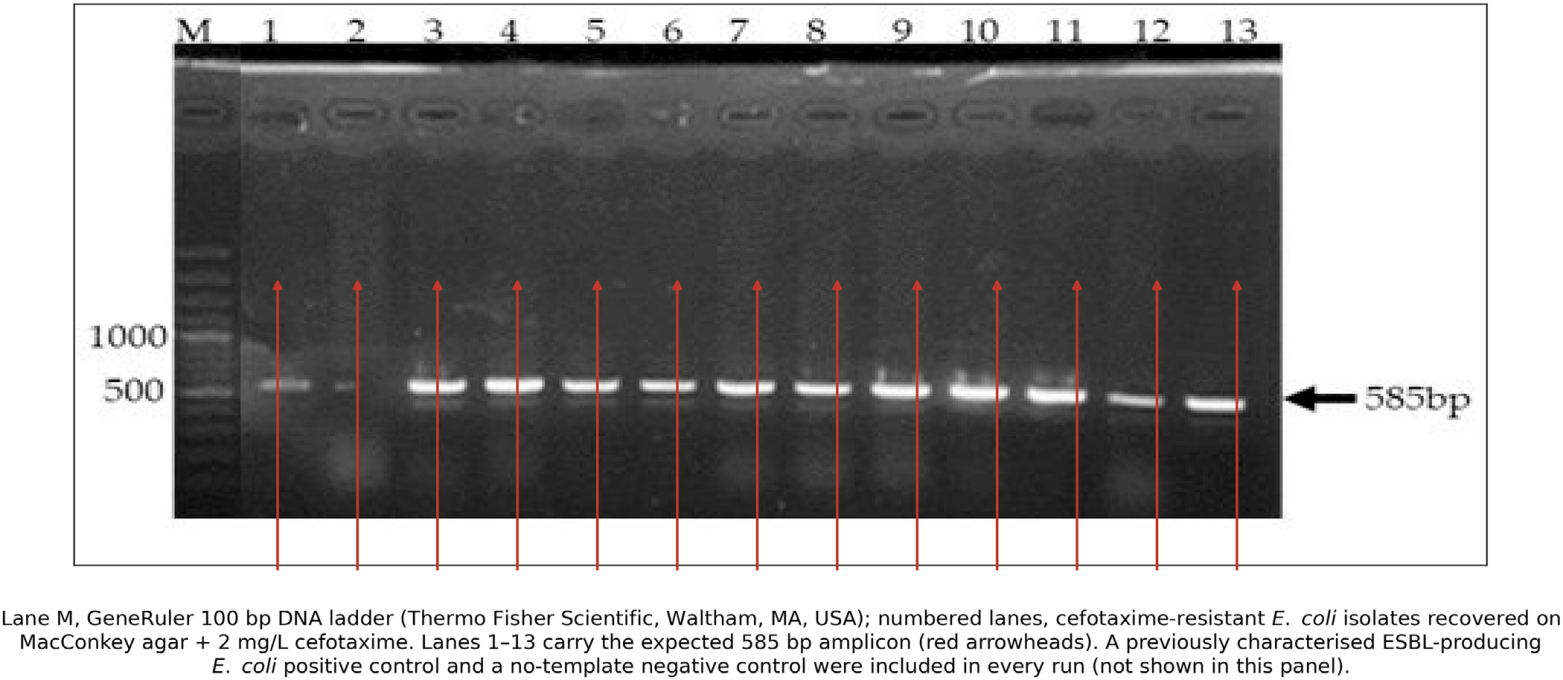
Representative agarose gel electrophoresis (1%, w/v) of the *bla_CTX‐M_* multiplex PCR product (expected amplicon size 585 bp). Lane M: 100 bp DNA ladder; Lane 1: positive control; Lane 2: no‐template (negative) control; Lanes 3–13: *bla_CTX‐M_*‐positive isolates (nos. 2, 4, 5, 6, 10, 15, 16, 17, 20, 21 and 23).

All 13 phenotypically confirmed ESBL isolates harboured *bla_CTX‐M_* (100% of ESBL isolates; 11.6% of all 112 samples, 95% CI: 6.9%–18.9%). *bla_TEM_* was detected in four isolates (30.8% of ESBL isolates; 3.6% of all samples, 95% CI: 1.4%–8.8%) and *bla_SHV_* in a single isolate (7.7% of ESBL isolates; 0.9% of all samples, 95% CI: 0.2%–4.9%; Table 5). Gene co‐occurrence was observed: *bla_CTX‐M_* + *bla_TEM_* in four isolates (3.6% of all samples) and *bla_CTX‐M_* + *bla_SHV_* in one isolate (0.9%); one isolate carried all three genes (0.9%). Representative gel images for *bla_CTX‐M_* (585 bp), *bla_TEM_* (1080 bp) and *bla_SHV_* (930 bp) amplicons are shown in Figures 2, 3, 4. The remaining 99 samples (88.4%, 95% CI: 81.1%–93.1%) were negative for all three target genes under the conditions employed.

**TABLE 5:** Distribution of ESBL genes among phenotypically confirmed ESBL‐producing *Escherichia coli* isolates (*n* = 13) and in relation to all samples (*n* = 112).

Gene profile	Isolates (*n*)	% of ESBL isolates (95% Wilson CI)	% of all 112 samples (95% Wilson CI)
*bla_CTX‐M_*	13	100.0 (77.2–100.0)	11.6 (6.9–18.9)
*bla_TEM_*	4	30.8 (12.7–57.6)	3.6 (1.4–8.8)
*bla_SHV_*	1	7.7 (1.4–33.3)	0.9 (0.2–4.9)
*bla_CTX‐M_* + *bla_TEM_*	4	30.8 (12.7–57.6)	3.6 (1.4–8.8)
*bla_CTX‐M_* + *bla_SHV_*	1	7.7 (1.4–33.3)	0.9 (0.2–4.9)
*bla_CTX‐M_* + *bla_TEM_* + *bla_SHV_*	1	7.7 (1.4–33.3)	0.9 (0.2–4.9)
No ESBL gene detected	99	— (—)	88.4 (81.1–93.1)

*Note*: Counts refer to isolates carrying each gene or gene combination; co‐occurrence is tabulated non‐exclusively (the single isolate carrying all three genes is counted in each pairwise row and in the triple‐carriage row). Percentages are given both relative to the 13 phenotypically confirmed ESBL isolates and relative to the 112 pooled samples analysed. 95% Wilson score confidence intervals are reported.

Abbreviations: CI, confidence interval; ESBL, extended‐spectrum *β*‐lactamase.

## 4 Discussion

This study provides the first comparative, molecularly supported estimate of the carriage of ESBL‐producing *E. coli* in intensively reared and free‐range chickens in Zambia. Three findings are salient. First, ESBL *E. coli* was detected in 11.6% of pooled flock‐level samples, a figure that sits within the broad range reported from poultry studies across sub‐Saharan Africa (Falgenhauer et al. 2019; Ojo et al. 2016). Second, prevalence was essentially identical in the two production systems (12.5% vs. 10.7%; *p* = 1.00), suggesting that the binary classification of ‘intensive’ versus ‘free‐range’ does not, on its own, predict the risk of ESBL carriage in this setting. Third, *bla_CTX‐M_* was the near‐universal genetic determinant, mirroring the global pan‐geographic dominance of CTX‐M enzymes among ESBL‐producing *Enterobacterales* (Bevan et al. 2017; Cantón et al. 2012).

The absence of a statistically detectable difference between production systems should be interpreted with caution given the modest number of ESBL‐positive flocks (*n* = 13) and the consequent wide CIs; the study is more informative as a baseline, hypothesis‐generating survey than as a definitive equivalence study. Nonetheless, the observation that free‐range flocks—which in our study had little reported direct exposure to antimicrobials—carried ESBL *E. coli* at a level comparable with intensive flocks echoes results from Ghana (Eibach et al. 2018), Bangladesh (Hasan and Swedberg 2022) and Europe (Kola et al. 2012; Huizinga et al. 2019), and is consistent with the hypothesis that environmental reservoirs, human‐mediated movement of birds and feed, and horizontal gene transfer sustain ESBL carriage even where on‐farm antimicrobial use is low (Koutsoumanis et al. 2021; von Wintersdorff et al. 2016).

All 13 ESBL isolates were multidrug‐resistant, with particularly high resistance to tetracycline (100%), streptomycin (84.6%) and sulfamethoxazole–trimethoprim (69.2%). Universal tetracycline resistance is consistent with the widespread availability and low cost of tetracyclines for poultry treatment and prophylaxis in Zambia and the region (Mwansa et al. 2022), and with the common physical linkage of *tet* genes to ESBL‐encoding plasmids (Carattoli 2013). The high rate of sulphonamide–trimethoprim resistance likewise mirrors reports from Tanzania and elsewhere in eastern and southern Africa (Ulomi et al. 2022). The retention of full ceftriaxone susceptibility by all isolates, alongside universal cefotaxime resistance, may reflect heterogeneity in CTX‐M enzyme subtypes (not further characterised here) and/or the use of the CLSI cefotaxime screening breakpoint on the initial isolation plate; subtyping by sequencing is a priority for follow‐up.

The exclusive detection of *bla_CTX‐M_* in 100% of ESBL isolates, with *bla_TEM_* and *bla_SHV_* playing only secondary, often co‐occurring, roles, is in close agreement with studies of poultry *E. coli* from Ghana (Eibach et al. 2018), India (Brower et al. 2017) and Europe (Kola et al. 2012; Huizinga et al. 2019). One isolate harboured all three genes (*bla_CTX‐M_*, *bla_TEM_* and *bla_SHV_*), a profile that has been associated with high‐risk *E. coli* clones such as ST131 in humans (Nicolas‐Chanoine et al. 2014) and warrants further molecular investigation.

From a One Health perspective, the similar ESBL‐carriage rates in the two rearing systems examined here indicate that interventions targeting only large‐scale intensive producers will be insufficient. Surveillance and stewardship must extend to village (free‐range) flocks, which account for a substantial share of Zambian household protein consumption, and must be integrated with environmental and human‐health surveillance, in keeping with the Tricycle framework (Puspandari et al. 2021).

### 4.1 Limitations

Two limitations merit particular emphasis.

First, the genotypic characterisation was restricted to the three most frequently reported ESBL gene families (*bla_CTX‐M_*, *bla_TEM_*, *bla_SHV_*). Important resistance determinants that also confer reduced susceptibility to *β*‐lactams—including *bla_OXA_*, *bla_CMY_* and other plasmid‐mediated AmpC genes, and carbapenemases such as *bla_NDM_*, *bla_KPC_* and *bla_OXA‐48_*—were not targeted, so the resistome captured in this study is necessarily incomplete. In addition, CTX‐M variant typing (e.g., CTX‐M‐15 vs. CTX‐M‐14) was not undertaken. *Future studies* should use either a broader multiplex panel (ideally including *bla_OXA_*, plasmid‐mediated AmpC genes and the principal carbapenemases), or, preferably, whole‐genome sequencing with resistome analysis via curated databases such as the Comprehensive Antibiotic Resistance Database (CARD) or ResFinder. Sequencing would additionally provide CTX‐M subtype, sequence type (ST) and plasmid context, all of which are essential for inferring transmission dynamics.

Second, sampling was restricted to two districts in Central Province and to a single cross‐sectional time point, and used pooled faecal samples of 10 birds per farm. This design efficiently estimates flock‐level prevalence but limits within‐flock inference and generalisability to other Zambian provinces and seasons; furthermore, farm‐level antimicrobial‐use data were not collected at sufficient granularity to allow formal risk‐factor modelling. *Future studies* should adopt a multi‐province, repeated cross‐sectional or longitudinal design with individual (rather than pooled) sampling where feasible, parallel collection of quantitative on‐farm antimicrobial‐use and biosecurity data and concurrent environmental (water, litter, feed) and human (farmer/handler) sampling to enable One Health inference. Increasing the overall sample size would also improve statistical power to detect small but epidemiologically meaningful differences between production systems.

## 5 Conclusions

Multidrug‐resistant, *bla_CTX‐M_*‐driven ESBL‐producing *E. coli* is present at comparable levels in both intensively reared and free‐range chickens in Kabwe and Kapiri Mposhi districts of Zambia (12.5% vs. 10.7%; *p* = 1.00), with blaCTX‐M detected in all 13 ESBL isolates. These findings challenge the assumption that free‐range poultry represent a low‐risk reservoir, and underscore the need for One Health surveillance and antimicrobial stewardship that encompasses both commercial and smallholder poultry sectors. Strengthening farmer education, enforcing prudent antimicrobial use and withdrawal periods, and integrating animal, human and environmental surveillance are urgent priorities for Zambia's National Action Plan on AMR.

## Author Contributions

Conceptualisation: Kenneth Chawinga, Isaac Thom Shawa, Rajhab Sawasawa Mkakosya, and Bernard M. Hang'ombe. Methodology: Kenneth Chawinga, Bernard M. Hang'ombe, Geoffrey Mainda, and Musso Munyeme. Formal analysis: Kenneth Chawinga and Isaac Thom Shawa. Investigation: Kenneth Chawinga, Francis Mbaimbai, Liywalii Mataa, Emmanuel Kabwali, and Fusya Y. Goma. Resources: Bernard M. Hang'ombe, Geoffrey Muuka, and Geoffrey Mainda. Data curation: Kenneth Chawinga and Liywalii Mataa. Writing – original draft: Kenneth Chawinga. Writing – review and editing: Isaac Thom Shawa, Bernard M. Hang'ombe, Geoffrey Mainda, Francis Mbaimbai, Liywalii Mataa, Emmanuel Kabwali, Fusya Y. Goma, Geoffrey Muuka, Musso Munyeme, and Rajhab Sawasawa Mkakosya. Supervision: Bernard M. Hang'ombe, Rajhab Sawasawa Mkakosya, and Isaac Thom Shawa. Project administration: Kenneth Chawinga.

## Funding

This work was supported by the World Bank through the Africa Centre of Excellence in Public and Herbal Medicine (ACEPHEM) at Kamuzu University of Health Sciences and the Africa Centre of Excellence for Infectious Diseases of Humans and Animals (ACEIDHA) at the University of Zambia, School of Veterinary Medicine (Grant number P151847). The funders had no role in study design, data collection, analysis, interpretation of data, writing of the manuscript or the decision to submit for publication.

## Ethics Statement

The study was approved by the Univerity of Malawi, College of Medicine Research Ethics Committee (COMREC; Approval No. P.08/20/3116; 11 November 2020) and conducted in accordance with the Declaration of Helsinki. Samples were obtained non‐invasively from freshly voided faeces of apparently healthy birds with the informed verbal consent of the flock owners. The study adhered to the ARRIVE 2.0 guidelines.

The study was conducted in accordance with the Declaration of Helsinki and was reviewed and approved by an independent Research Ethics Committee. Faecal samples were collected non‐invasively from freshly voided droppings of apparently healthy birds with the informed verbal consent of the flock owner; no handling, restraint or invasive procedures were performed on animals. The study adhered to the ARRIVE 2.0 guidelines for reporting research involving animals where applicable.

## Conflicts of Interest

The authors declare no conflicts of interest.

## Supporting information




**Supporting File**: vms371090‐supp‐0001‐SuppMat.xlsx

## Data Availability

The raw dataset generated during the current study is provided as Supporting Information accompanying this article (Poultry Survey Raw Data 2021). Any further information is available from the corresponding author upon reasonable request.

## References

[vms371090-bib-0001] Agyare, C. , V. E. Boamah , C. Ngofi Zumbi , and F. B. Osei . 2019. “Antibiotic Use in Poultry Production and Its Effects on Bacterial Resistance.” In Antimicrobial Resistance—A Global Threat. IntechOpen. 10.5772/intechopen.79371.

[vms371090-bib-0002] Ahmed, S. K. , S. Hussein , K. Qurbani , et al. 2024. “Antimicrobial Resistance: Impacts, Challenges, and Future Prospects.” Journal of Medicine, Surgery, and Public Health 2: 100081. 10.1016/j.glmedi.2024.100081.

[vms371090-bib-0003] Bevan, E. R. , A. M. Jones , and P. M. Hawkey . 2017. “Global Epidemiology of CTX‐M β‐Lactamases: Temporal and Geographical Shifts in Genotype.” Journal of Antimicrobial Chemotherapy 72, no. 8: 2145–2155. 10.1093/jac/dkx146. 28541467

[vms371090-bib-0004] Brower, C. H. , S. Mandal , S. Hayer , et al. 2017. “The Prevalence of Extended‐Spectrum Beta‐Lactamase‐Producing Multidrug‐Resistant *Escherichia coli* in Poultry Chickens and Variation According to Farming Practices in Punjab, India.” Environmental Health Perspectives 125, no. 7: 077015. 10.1289/EHP292. 28749780 PMC5744676

[vms371090-bib-0005] Cantón, R. , J. M. González‐Alba , and J. C. Galán . 2012. “CTX‐M Enzymes: Origin and Diffusion.” Frontiers in Microbiology 3: 110. 10.3389/fmicb.2012.00110. 22485109 PMC3316993

[vms371090-bib-0006] Carattoli, A. 2013. “Plasmids and the Spread of Resistance.” International Journal of Medical Microbiology 303, no. 6–7: 298–304. 10.1016/j.ijmm.2013.02.001. 23499304

[vms371090-bib-0007] Chileshe, C. , M. Shawa , N. Phiri , et al. 2024. “Detection of Extended‐Spectrum Beta‐Lactamase (ESBL)‐Producing Enterobacteriaceae From Diseased Broiler Chickens in Lusaka District, Zambia.” Antibiotics 13, no. 3: 259. 10.3390/antibiotics13030259. 38534694 PMC10967349

[vms371090-bib-0008] Clinical and Laboratory Standards Institute . 2020. “Performance Standards for Antimicrobial Susceptibility Testing.” In CLSI Supplement M100. 30th ed. CLSI.

[vms371090-bib-0009] Dashti, A. A. , M. M. Jadaon , A. M. Abdulsamad , and H. M. Dashti . 2009. “Heat Treatment of Bacteria: A Simple Method of DNA Extraction for Molecular Techniques.” Kuwait Medical Journal 41, no. 2: 117–122.

[vms371090-bib-0010] De Carli, S. , N. Ikuta , F. K. M. Lehmann , et al. 2015. “Virulence Gene Content in *Escherichia coli* Isolates From Poultry Flocks With Clinical Signs of Colibacillosis in Brazil.” Poultry Science 94, no. 11: 2635–2640. 10.3382/ps/pev256. 26371329

[vms371090-bib-0011] Dierikx, C. M. , J. A. van der Goot , H. E. Smith , A. Kant , and D. J. Mevius . 2013. “Presence of ESBL/AmpC‐Producing *Escherichia coli* in the Broiler Production Pyramid: A Descriptive Study.” PLoS ONE 8, no. 11: e79005. 10.1371/journal.pone.0079005. 24244401 PMC3820706

[vms371090-bib-0012] Eibach, D. , D. Dekker , K. Gyau Boahen , et al. 2018. “Extended‐Spectrum Beta‐Lactamase‐Producing *Escherichia coli* and *Klebsiella pneumoniae* in Local and Imported Poultry Meat in Ghana.” Veterinary Microbiology 217: 7–12. 10.1016/j.vetmic.2018.02.023. 29615260

[vms371090-bib-0013] Erkmen, O. 2021. “Pure Culture Techniques.” In Laboratory Practices in Microbiology. Academic Press. 10.1016/b978-0-323-91017-0.00002-0.

[vms371090-bib-0014] Falgenhauer, L. , C. Imirzalioglu , K. Oppong , et al. 2019. “Detection and Characterization of ESBL‐Producing *Escherichia coli* From Humans and Poultry in Ghana.” Frontiers in Microbiology 10: 3358. 10.3389/fmicb.2018.03358. PMC6340976 30697208

[vms371090-bib-0015] Gonzalez Ronquillo, M. , and J. C. Angeles Hernandez . 2017. “Antibiotic and Synthetic Growth Promoters in Animal Diets: Review of Impact and Analytical Methods.” Food Control 72: 255–267. 10.1016/j.foodcont.2016.03.001.

[vms371090-bib-0016] Hasan, B. , and G. Swedberg . 2022. “Molecular Characterization of Clinically Relevant Extended‐Spectrum β‐Lactamases Bl_CTX‐M‐15_ ‐Producing Enterobacteriaceae Isolated From Free‐Range Chicken From Households in Bangladesh.” Microbial Drug Resistance 28, no. 7: 780–786. 10.1089/mdr.2021.0264. 35759384

[vms371090-bib-0017] Hedman, H. D. , K. A. Vasco , and L. Zhang . 2020. “A Review of Antimicrobial Resistance in Poultry Farming Within Low‐Resource Settings.” Animals 10, no. 8: 1264. 10.3390/ani10081264. 32722312 PMC7460429

[vms371090-bib-0018] Huizinga, P. , M. Kluytmans‐van den Bergh , J. W. Rossen , et al. 2019. “Decreasing Prevalence of Contamination With Extended‐Spectrum Beta‐Lactamase‐Producing Enterobacteriaceae (ESBL‐E) in Retail Chicken Meat in the Netherlands.” PLoS ONE 14, no. 12: e0226828. 10.1371/journal.pone.0226828. 31891609 PMC6938319

[vms371090-bib-0019] Jenkins, C. 2015. “Commentary: Whole‐Genome Sequencing Data for Serotyping *Escherichia coli*—It's Time for a Change!” Journal of Clinical Microbiology 53, no. 8: 2402–2403. 10.1128/JCM.01448-15. 26085609 PMC4508412

[vms371090-bib-0020] Kazemian, H. , H. Heidari , R. Ghanavati , et al. 2019. “Phenotypic and Genotypic Characterization of ESBL‐, AmpC‐, and Carbapenemase‐Producing *Klebsiella pneumoniae* and *Escherichia coli* Isolates.” Medical Principles and Practice 28, no. 6: 547–551. 10.1159/000500311. 30995662 PMC6944897

[vms371090-bib-0021] Kola, A. , C. Kohler , Y. Pfeifer , et al. 2012. “High Prevalence of Extended‐Spectrum‐ ‐Lactamase‐Producing Enterobacteriaceae in Organic and Conventional Retail Chicken Meat, Germany.” Journal of Antimicrobial Chemotherapy 67, no. 11: 2631–2634. 10.1093/jac/dks295. 22868643

[vms371090-bib-0022] Koutsoumanis, K. , A. Allende , A. Álvarez‐Ordóñez , et al. 2021. “Role Played by the Environment in the Emergence and Spread of Antimicrobial Resistance (AMR) Through the Food Chain.” EFSA Journal 19, no. 6: 6651. 10.2903/j.efsa.2021.6651. PMC8210462 34178158

[vms371090-bib-0023] Magiorakos, A.‐P. , A. Srinivasan , R. B. Carey , et al. 2012. “Multidrug‐Resistant, Extensively Drug‐Resistant and Pandrug‐Resistant Bacteria: An International Expert Proposal for Interim Standard Definitions for Acquired Resistance.” Clinical Microbiology and Infection 18, no. 3: 268–281. 10.1111/j.1469-0691.2011.03570.x. 21793988

[vms371090-bib-0024] Mahmud, Z. H. , M. H. Kabir , S. Ali , et al. 2020. “Extended‐Spectrum Beta‐Lactamase‐Producing *Escherichia coli* in Drinking Water Samples From a Forcibly Displaced, Densely Populated Community Setting in Bangladesh.” Frontiers in Public Health 8: 228. 10.3389/fpubh.2020.00228. 32626677 PMC7314906

[vms371090-bib-0025] Ministry of Fisheries and Livestock . 2023. 2021 Livestock and Aquaculture Census Report. Zambia Statistical Agency (Zamstats). https://www.zamstats.gov.zm/publications/.

[vms371090-bib-0026] Musaba, E. C. , and M. Mseteka . 2014. “Cost Efficiency of Small‐Scale Commercial Broiler Production in Zambia: A Stochastic Cost Frontier Approach.” Developing Country Studies 4, no. 5: 14–21.

[vms371090-bib-0027] Mwansa, T. N. , K. Kamvuma , J. A. Mulemena , C. N. Phiri , and W. Chanda . 2022. “Antibiotic Susceptibility Patterns of Pathogens Isolated From Laboratory Specimens at Livingstone Central Hospital in Zambia.” PLOS Global Public Health 2, no. 9: e0000623. 10.1371/journal.pgph.0000623. 36962542 PMC10022373

[vms371090-bib-0028] Nene, M. , N. W. Kunene , R. Pierneef , and K. Hadebe . 2024. “Profiling the Diversity of the Village Chicken Faecal Microbiota Using 16S rRNA Gene and Metagenomic Sequencing Data to Reveal Patterns of Gut Microbiome Signatures.” Frontiers in Microbiology 15: 1487595. 10.3389/fmicb.2024.1487595. 39968048 PMC11832711

[vms371090-bib-0029] Nicolas‐Chanoine, M. H. , X. Bertrand , and J. Y. Madec . 2014. “ *Escherichia coli* ST131, an Intriguing Clonal Group.” Clinical Microbiology Reviews 27, no. 3: 543–574. 10.1128/CMR.00125-13. 24982321 PMC4135899

[vms371090-bib-0030] Ojo, O. E. , S. Schwarz , and G. B. Michael . 2016. “Detection and Characterization of Extended‐Spectrum β‐Lactamase‐Producing *Escherichia coli* From Chicken Production Chains in Nigeria.” Veterinary Microbiology 194: 62–68. 10.1016/j.vetmic.2016.04.022. 27157499

[vms371090-bib-0031] Persoons, D. , K. Bollaerts , A. Smet , et al. 2011. “The Importance of Sample Size in the Determination of a Flock‐Level Antimicrobial Resistance Profile for *Escherichia coli* in Broilers.” Microbial Drug Resistance 17, no. 4: 513–519. 10.1089/mdr.2011.0048. 21875337 PMC3223426

[vms371090-bib-0032] Phiri, N. , G. Mainda , M. Mukuma , et al. 2020. “Antibiotic‐Resistant *Salmonella* Species and *Escherichia coli* in Broiler Chickens From Farms, Abattoirs, and Open Markets in Selected Districts of Zambia.” Journal of Epidemiological Research 6, no. 1: 13. 10.5430/jer.v6n1p13.

[vms371090-bib-0033] Puspandari, N. , S. Sunarno , T. Febrianti , et al. 2021. “Extended Spectrum Beta‐Lactamase‐Producing *Escherichia coli* Surveillance in the Human, Food Chain, and Environment Sectors: Tricycle Project (Pilot) in Indonesia.” One Health 13: 100331. 10.1016/j.onehlt.2021.100331. 34632041 PMC8493575

[vms371090-bib-0034] Ranjan, A. , S. Shaik , A. Hussain , et al. 2015. “Genomic and Functional Portrait of a Highly Virulent, CTX‐M‐15‐Producing H 30‐Rx Subclone of *Escherichia coli* Sequence Type 131.” Antimicrobial Agents and Chemotherapy 59, no. 10: 6087–6095. 10.1128/AAC.01447-15. 26195517 PMC4576125

[vms371090-bib-0035] Rouger, A. , O. Tresse , and M. Zagorec . 2017. “Bacterial Contaminants of Poultry Meat: Sources, Species, and Dynamics.” Microorganisms 5, no. 3: 50. 10.3390/microorganisms5030050. 28841156 PMC5620641

[vms371090-bib-0036] Ulomi, W. J. , F. X. Mgaya , Z. Kimera , and M. I. Matee . 2022. “Determination of Sulphonamides and Tetracycline Residues in Liver Tissues of Broiler Chicken Sold in Kinondoni and Ilala Municipalities, Dar es Salaam, Tanzania.” Antibiotics 11, no. 9: 1222. 10.3390/antibiotics11091222. 36140001 PMC9495219

[vms371090-bib-0037] van den Bogaard, A. E. , and E. E. Stobberingh . 2001. “Antibiotic Resistance of Faecal *Escherichia coli* in Poultry, Poultry Farmers and Poultry Slaughterers.” Journal of Antimicrobial Chemotherapy 47, no. 6: 763–771. 10.1093/jac/47.6.763. 11389108

[vms371090-bib-0038] von Wintersdorff, C. J. H. , J. Penders , J. M. van Niekerk , et al. 2016. “Dissemination of Antimicrobial Resistance in Microbial Ecosystems Through Horizontal Gene Transfer.” Frontiers in Microbiology 7: 173. 10.3389/fmicb.2016.00173. 26925045 PMC4759269

[vms371090-bib-0039] World Health Organization . 2017. Zambia National Action Plan on Antimicrobial Resistance. WHO.

[vms371090-bib-0040] Ziba, M. W. , B. Bowa , R. Romantini , et al. 2020. “Occurrence and Antimicrobial Resistance of *Salmonella* spp. in Broiler Chicken Neck Skin From Slaughterhouses in Zambia.” Journal of Veterinary Medicine and Animal Health 12, no. 4: 142–149.

